# Bladder Cancer in an Inguinoscrotal Vesical Hernia

**DOI:** 10.1155/2012/142351

**Published:** 2012-10-18

**Authors:** Lucas Regis, Fernando Lozano, Jacques Planas, Juan Morote

**Affiliations:** Servicio de Urología, Hospital General Universitario Vall d'Hebron, Pg. De la Vall d'Hebron 119-129, 08035 Barcelona, Spain

## Abstract

We present the case of a 79-year-old male who, due to hematuria, underwent cystoscopy that showed a lesion in the bladder dome. Transurethral resection was attempted, but access to the tumor by this route was impossible. Given the findings, a body CT scan was performed showing an inguinoscrotal hernia with vesical carcinoma contained. Open surgical treatment of the vesical carcinoma contained within the inguinoscrotal hernia was performed in conjunction with the hernia repair. The anatomical pathology report confirmed a high-grade urothelial carcinoma (stage pT2b) with a free resection margin of <1 mm. Adjuvant radiotherapy was selected for subsequent treatment. The presence of bladder tumor in an inguinoscrotal hernia is an uncommon finding and a diagnostic delay can be assumed. The initial therapeutic plan may need to be changed from the usual approaches due to the atypical presentation.

## 1. Introduction

The presence of the bladder wall is an infrequent finding in inguinal hernias, occurring in fewer than 4% of cases, according to published series [1, 2]. The presence of bladder cancer within such a herniation is an anecdotal finding that has been described in isolated cases in the literature [3]. We present the case of a patient with a bladder carcinoma within an inguinovesical hernia that was not diagnosed prior to the herniation (its atypical presentation was discovered during the surgery.)

## 2. Case Presentation

We present the case of a 79-year-old male with type II diabetes, hypertension, dyslipidemia, a history of transient ischemic attacks, and chronic renal insufficiency due to vascular nephropathy. He had been followed by the urology service for the previous 2 years due to irritative voiding symptoms with normal PSA and physical examination. Due to an episode of macroscopic hematuria, imaging studies—ultrasound and computer-aided tomography (CT)—were performed without significant findings. Therefore, urethroscopy was chosen to aid the etiological diagnosis. A 1.5 cm multilobulated lesion, with a solid appearance suggestive of bladder cancer and positive cytology for malignant cells was appreciated at the dome of the bladder.

**Figure 1:**
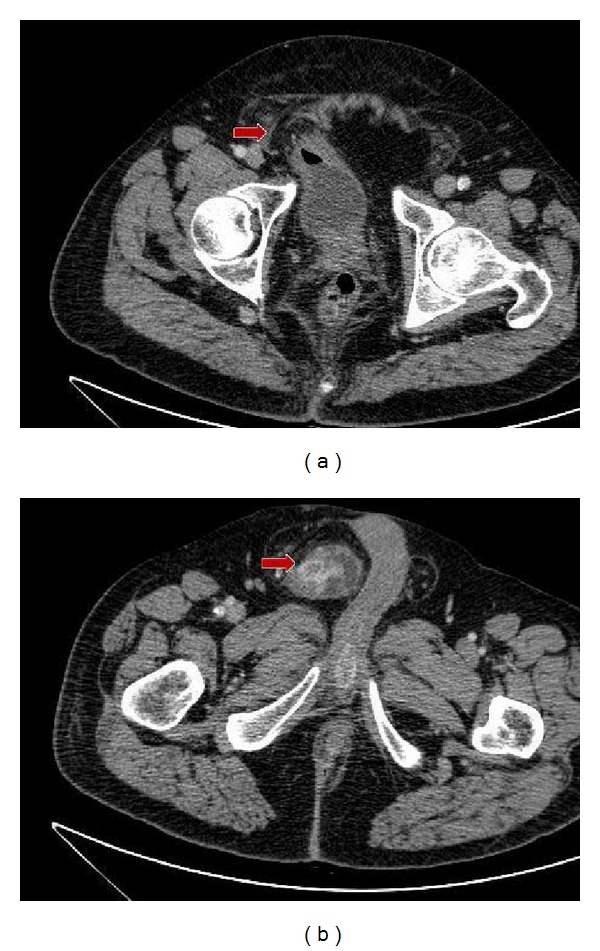
A right inguinoscrotal hernia with bladder contents. Note that the image suggests a bladder tumor in the 3.5 cm hernia sac.

**Figure 2:**
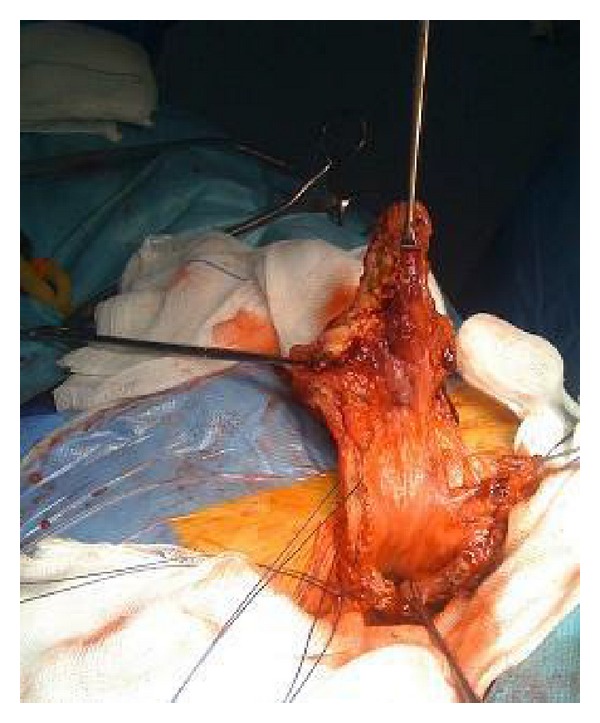
An intraoperative view of the herniated portion of the bladder.

Excision of the lesion via TUR was attempted during the surgery, but it was impossible to access the tumor using the resector due to access difficulties. The tumor was visualized in the dome and anterior wall adjacent to an apparent diverticular region that could not be passed by the resectoscope. Given the findings, a body CT scan was performed showing a bladder tumor located in an inguinovesical herniation (Figures 1(a) and 1(b): a right inguinoscrotal hernia with bladder contents. Note that the image suggests a bladder tumor in the 3.5 cm hernia sac). Therefore, an additional procedure was performed. Under epidural anesthesia, the herniation was manually reduced intraoperatively, which allowed access to the portion of the bladder that contained the neoplasm. A partial cystectomy and herniorrhaphy were performed, and free margins of macroscopic evidence of disease were obtained (Figure 2: An intraoperative view of the herniated portion of the bladder). The anatomical pathology report confirmed a high-grade urothelial carcinoma with pT2b glandular differentiation, 10% squamous differentiation, a maximum diameter of 4.5 cm, and free resection margins of <1 mm. Adjuvant radiotherapy was selected for subsequent treatment.

## 3. Discussion

The presence of the bladder wall is an infrequent finding in inguinal hernias, although prevalences of up to 10% have been described in patients older than 50 years old. The following factors that contribute to the pathogenesis of this process are particularly notable: weakness of the abdominal wall, obesity, lower urinary tract obstruction, changes in bladder morphology as a consequence of higher bladder pressure and prior surgeries or local trauma [1, 2].

Vesical herniation is most frequently (75%) associated with inguinal hernias; it is linked to crural hernias in 23% of cases and to other types (such as incisional, umbilical, and obturator) in 2% [4]. Classically, bladder protrusion through a hernia orifice has been classified into three major groups based on the relationship between the herniated portion of the bladder and the parietal peritoneum: paraperitoneal, extraperitoneal, and intraperitoneal [2, 5]. The intraperitoneal form, in which the dome and the posterior wall constitute the herniated portion and are completely wrapped by the peritoneum, is the least common (4%). Extraperitoneal hernias (32%) tend to be small and are not covered by the peritoneum. Paraperitoneal hernias, which are covered by the peritoneum only on the external face of the herniated bladder, are the most common. Paraperitoneal hernias are voluminous and have a prevalence of approximately 60% [2].

Our case was a right-sided paraperitoneal hernia, which is the most prevalent (60%) type [5]. Vesical hernias are initially asymptomatic in the vast majority of cases. When they are symptomatic, a characteristic divided-stream micturition in response to a manual scrotal pressure maneuver is the classic finding (known as the Mery sign) [1, 4–6]. The presence of bladder carcinoma in the herniation is an anecdotal finding that has been described in isolated cases in the literature [1, 3, 7]. Other manifestations associated with this entity that have been described in the literature include vesicoureteral reflux, renal insufficiency, and lithiasis [6].

Voiding urethrocistoscopy has been considered to be the test of choice due to its higher sensitivity for diagnosing bladder hernias [5, 6]. Some authors have even recommended it for all patients over 50 years of age who present with large-sized hernias at diagnosis. Others have emphasized the role of ultrasound in the initial diagnosis due to its cost effectiveness and noninvasive nature. As for the intravenous urography findings, Reardon and Lowman have described the classic triad of this nosologic entity (lateral displacement of the ureter, a small/asymmetric bladder and an incompletely visualized bladder base). Cystoscopy provides additional information about the herniated contents, such as lithiasis and tumor. Other more sophisticated imaging techniques, such as helical CT and MRI, have the capacity to evaluate the vesicoureteral morphology of each case through plane reconstruction, which has certain advantages. Moreover, these diagnostic modalities permit visualizing the hernia contents and further study of possible intrahernial neoplasic processes [6–8].

The presence of malignant neoplasms in inguinal hernias is an infrequent finding, occurring in less than 0.5% of the herniorrhaphies described in the literature [6, 9].

The most frequent etiology is metastatic tumors, the vast majority of which originate in the gastrointestinal tract. Other possibilities include aggressive mesothelial, ovarian, hematological and vesical sarcomas and angiomyxomas, melanomas, endometrial cancer and tumors of the thymus, kidney, and pericardium. According to the literature, urothelial carcinoma of the herniated bladder account for 5.34% of hernia sac tumors [9].

Some authors have described a poor survival rate (caused by diagnostic delay) in patients with tumor of the herniated portion of the bladder [9]. Studies of the prognostic factors for carcinomas contained in vesical diverticulum show that the prognosis seems to depend solely on the clinical and anatomic pathological staging [10]. Similar studies of carcinomas in the herniated portion of the bladder are necessary to determine the real associations between the atypical presentation and its survival rates.
